# Advancing Nutrient Management Strategies for Sustainable Crop Productivity in a Changing Climate: A Systematic Review

**DOI:** 10.1155/tswj/7101060

**Published:** 2025-11-14

**Authors:** Arebu Hussen Yimer, Akos Tarnawa

**Affiliations:** ^1^Institute of Agronomy, Doctoral School of Plant Science, Hungarian University of Agriculture and Life Sciences (MATE), Gödöllo, Hungary; ^2^Department of Plant Science, College of Agriculture and Natural Resources, Mekdela Amba University, Tulu Awulia, Ethiopia

**Keywords:** climate change, crop productivity, nutrient management, precision agriculture, soil fertility, sustainability

## Abstract

Climate change poses significant challenges to global food security by disrupting agricultural nutrient dynamics through increased temperatures, altered precipitation patterns, and extreme weather events. These changes threaten crop productivity, soil health, and environmental sustainability. Traditional nutrient management practices, often reliant on excessive chemical fertilizer use, contribute to nutrient losses, soil degradation, and greenhouse gas emissions. This review systematically analyzes 65 peer-reviewed studies (1998–2024) selected using PRISMA guidelines, supplemented by bibliometric tools, to evaluate nutrient management strategies under climate change. The results highlight climate change's multifaceted impacts on soil nutrient cycles, microbial activity, crop physiology, and crop yield. Elevated temperatures and CO_2_ levels alter nutrient availability and reduce grain quality, while erratic rainfall patterns exacerbate nutrient losses through leaching and runoff. Conventional fertilizer practices are shown to be inefficient and environmentally harmful, prompting a shift toward integrated nutrient management, precision agriculture, and biofertilizers. Emerging strategies such as slow- and controlled-release fertilizers, site-specific nutrient management, and decision support systems significantly improve nutrient use efficiency and reduce greenhouse gas emissions. Conservation agriculture and organic amendments further enhance soil health and resilience. The discussion highlights that integrated and adaptive nutrient management frameworks, supported by technology and agroecological practices, are critical for maintaining high productivity while minimizing environmental impacts under climate change. These approaches collectively support sustainable crop production, mitigate climate impacts, and promote long-term soil fertility. The review concludes that nutrient management is central to climate-smart agriculture and offers actionable insights for researchers, farmers, and policymakers aiming to secure food systems in a changing climate.

## 1. Introduction

The challenge of ensuring global food security in the face of escalating climate change is one of the most pressing issues of our time. Climate change poses important challenges to agriculture, including increased temperatures, altered precipitation patterns, and more frequent extreme weather events. The delicate balance of nutrient dynamics in agricultural systems, critical for crop productivity, is increasingly threatened by shifting climatic conditions [1]. These changes can lead to reduced crop yields, increased pest and disease pressures, and soil degradation. Traditional farming practices often use excessive chemical fertilizers, causing nutrient runoff, water pollution, and soil degradation. Sustainable nutrient management, known as the 4Rs of nutrient stewardship, focuses on the right source, rate, time, and place for application, ensuring efficient crop nutrient delivery and reducing waste and environmental harm [2, 3]. Balanced irrigation and nutrient management practices enhance crop nutrient uptake, minimize ecological losses, and reduce greenhouse gas (GHG) emissions, thereby reducing environmental footprints [4]. Nutrient management strategies are crucial in agriculture for optimizing nutrient use, enhancing crop productivity, and minimizing environmental impacts. They involve the careful application of fertilizers and soil amendments to maintain soil health, improve crop yields, and promote sustainable agricultural practices [5, 6]. As Zhang et al. [7] report, the need for new advances in agricultural practices to ensure sustainability over the next 50 years is evident. It advocates for a holistic approach that includes optimizing nutrient inputs, reducing nitrogen (N) losses, and enhancing soil quality through practices such as returning straw to soils and conservation tillage. Nutrient-efficient plants are expected to play a crucial role in raising crop yields in the 21^st^ century. This is particularly important given the challenges of finite land and water resources, rising fertilizer costs, and environmental concerns [8]. Farmers can enhance fertilizer utilization efficiency (FUE) by implementing strategies like nutrient management planning, precision agriculture techniques, improved fertilizer formulations, and nutrient stewardship practices, which minimize nutrient losses and reduce environmental impacts while maintaining agricultural productivity [9]. Proper nutrient management, including the balanced application of fertilizers, significantly boosts crop yields. For instance, the application of 100% NPK (nitrogen, phosphorus, and potassium) resulted in higher grain yields compared to imbalanced nutrient applications, which led to lower productivity [10]. Banding application of phosphorus (P) with sulfuric acid results in higher P uptake and grain yield compared to broadcast methods, as well as effective P nutrient management in achieving optimal crop performance [11]. The use of advanced nutrient management practices helps in addressing the natural and temporal variability of nutrients in the soil. This approach is aimed at enhancing productivity and profitability while ensuring agricultural sustainability [3]. Adaptive nutrient management improves crop resilience to climate extremes, enhancing soil structure and water retention. Cover cropping and agroforestry mitigate drought and flood impacts, while site-specific nutrient management (SSNM) ensures optimal availability during critical growth stages [5].

Integrated nutrient management (INM) practices can reduce N losses and GHG emissions, contributing to environmental sustainability. This dual benefit of enhancing productivity while protecting the environment is a key takeaway [12]. The study by Kakraliya et al. [13] suggests that combining organic and inorganic fertilizers improves soil structure and water-holding capacity, thereby enhancing nutrient availability and promoting sustainable agricultural practices in the face of climate change. INM can increase crop yields by 8%–150% compared to conventional practices and highlights the effectiveness of INM in boosting agricultural productivity while maintaining sustainability [6, 12]. Parewa et al. [14] reported that using a combination of 75% NPK and 10 t ha^−1^ farmyard manure (FYM), along with bioinoculants, can achieve high productivity while reducing the reliance on chemical fertilizers as well as maintaining soil health and environmental sustainability. By promoting the utilization of organic fertilizers and biostimulants, the study encourages sustainable agricultural practices that can reduce reliance on chemical fertilizers; this shift can help mitigate environmental issues associated with chemical use, such as soil degradation and water pollution [15]. Advanced INM practices can significantly reduce reactive N losses and GHG emissions and mitigate eutrophication and soil degradation. This is particularly important as agriculture is a main contributor to non-CO_2_ GHG emissions, which have been on the rise due to synthetic fertilizer use. The study demonstrates how to improve nutrient capture from manure using a low-emission sliding shoe applicator on grass and precision injection of sludge into corn, thereby enhancing N and P management and reducing environmental impacts from nutrient runoff [16]. Improving nutrient use efficiency (NUE), especially for N, where only 30%–40% of applied N is typically utilized by the crop, is necessary. This inefficiency not only affects yield but also contributes to environmental issues, necessitating better management practices to minimize losses [2]. Nutrient management strategies enhance climate resilience by improving soil moisture retention and nutrient availability, making crops more resilient to droughts and extreme weather events. Biochar, for instance, improves water retention in soils, supporting crop growth during dry periods [17]. Therefore, the objectives of this review article are to examine how nutrient management strategies can improve yield, physiology, and grain quality under climate change; promote sustainable agricultural systems; identify knowledge gaps and challenges in crop nutrient management; and provide recommendations for future research and policy. The article can help to develop resilient crop production and serve as a valuable resource for agronomists, researchers, and farmers, allowing for better decision-making and advancements in field crop cultivation for improved food security and sustainable agricultural practices in the face of changing climates.

## 2. Methodology

### 2.1. Review Protocol

The full-text articles were searched to address the objective of this study based on the eligibility criteria (exclusion and inclusion criteria). Therefore, studies related to nutrient management strategies under climate change situations were critically identified by considering the intervention variable. Exclusion criteria were articles that were not written in English, articles published before 1998, book chapters, books, time series, and panel data types of articles, categorical measures for the intervention variable, articles that have no full information, and duplicated articles to maintain procedural similarities between studies throughout article browsing. Furthermore, the specified inclusion criteria included articles written in English that focused on field and greenhouse experiments, experimental trials, case studies, and long-term observational studies, all of which were included in the chosen papers from 1998 to 2024. The study followed the proposed checklists of the preferred reporting items for systematic reviews (PRISMA) guidelines for the screening process [18]. Using four-stage article selection procedures (i.e., identification, screening, eligibility, and inclusion), duplicate articles were removed. Then, through reading titles and/or abstracts, only relevant articles were screened. Finally, 65 appropriate full-text articles were included for data extraction.

### 2.2. Data Browsing Strategy

The article searching process was conducted systematically using electronic databases, such as Google Scholar, ScienceDirect, AGRIS, and ResearchGate, employing keywords and advanced search strategies with the aid of Boolean operators (AND/OR) to combine keywords. Moreover, data were retrieved from reference lists of eligible articles to increase the chance of getting relevant studies. Keywords like climate change and variability, nutrient management, fertilizer management, precision agriculture, sustainable agriculture, crop productivity, conventional farming, and soil fertility were used to get the required full-text articles that could respond to the review questions. The database search was conducted from November 15, 2024, to May 15, 2025.

### 2.3. Article Selection

**Figure 1:**
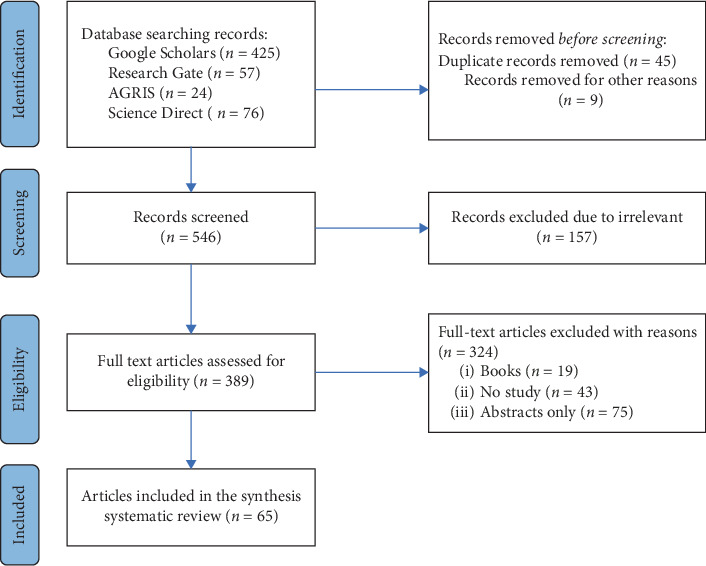
Flow diagram showing the article selection process.

As shown in Figure 1, using different database searching engines, a total of 425 studies from Google Scholar, 57 studies from ResearchGate, 24 studies from AGRIS, and 76 studies from ScienceDirect were initially recorded through the referencing of eligible studies. Finally, through applying different eligibility criteria and PRISMA guidelines, only 65 relevant studies were included for qualitative and quantitative analyses. Moreover, the selected articles used to conduct systematic reviews are shown in Table 1.

### 2.4. Data Extraction Process

The collected research articles were screened through the review of titles and abstracts for relevance to the research objectives of the review paper. This screening allowed for the exclusion of studies that did not meet the basic inclusion criteria and were removed from further consideration. To filter the selection further, we applied specific thematic criteria: “climate change impacts on nutrient dynamics and crop productivity,” “conventional nutrient management under climate change and their limitations,” and “the promising emerging nutrient management for achieving sustainable crop production in a climate-altered world and their challenges and opportunities.” Finally, applying this well-structured methodology ensured that only articles relevant to the objectives of the study were considered for deeper analysis for the study.

### 2.5. Statistical Analysis

A bibliometric analysis was also conducted using VOSviewer and the Bibliometrix R-package to evaluate key indicators such as publication patterns, influential authors, institutions, journals, and countries, along with coauthorship, citation, and keyword co-occurrence networks. Data spanning 1998–2024 were obtained from a recognized scientific database and examined to uncover research trends, collaboration structures, citation landscapes, and thematic clusters, thereby highlighting research hotspots and the evolution of themes in this field.

## 3. Results and Discussion

### 3.1. Climate Change Impacts on Nutrient Dynamics and Crop Productivity

**Figure 2:**
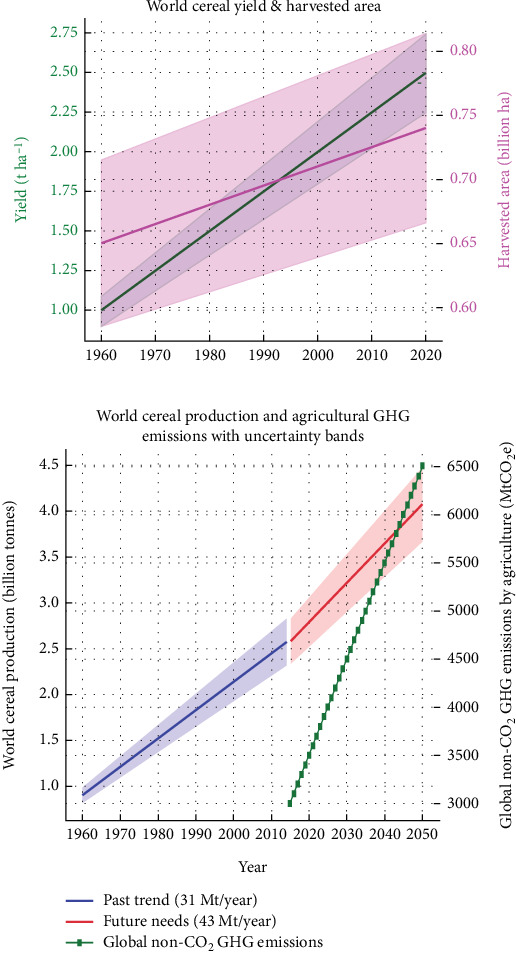
(a) The world cereal yield and harvested area and (b) world cereal production and agricultural GHG emissions with uncertainty bands [6].

Climate change impacts nutrient cycles and crop productivity in multifaceted ways. The interaction between climate change and agricultural systems is complex, with significant impacts on soil nutrient dynamics and crop productivity [19]. Climate change causes abiotic stresses on plants, affecting agricultural productivity. Global cereal production, particularly maize and wheat, is projected to decrease by 3.8% and 5.5%, necessitating a 60% increase by 2050, particularly in developing countries, to meet food and nutritional needs [20]. The required cereal production growth rate is 43 million tonnes/year, indicating a widening gap between supply and demand. Variability in past and future cereal production is due to weather, technology adoption, and policy support. Agricultural non-CO_2_ GHG emissions are projected to rise, stressing the environmental cost of intensification (Figure 2b). Past production gains were mainly from intensification, but future sustainability may require yield improvements and better resource management (Figure 2a,b).

#### 3.1.1. Effects of Temperature on Crop Yield and Nutrient Availability

Elevated temperatures can accelerate soil organic matter decomposition, leading to increased N mineralization and potentially higher nutrient availability [21]. However, this increased mineralization can also be coupled with increased volatilization of N gases, resulting in net losses of essential nutrients from the soil [22]. Higher temperatures accelerate soil organic matter mineralization and decomposition, reducing soil organic carbon pools and nutrient retention, while CO_2_ boosts plant growth and carbon sequestration; it also increases microbial activity, potentially leading to increased respiration rates and further soil organic carbon loss [23]. The review emphasizes the impact of rising temperatures and carbon dioxide on flowering time in crops and plants, highlighting the role of plant tissue temperature in determining anthesis time and suggesting replacing ambient air temperature with canopy or floral tissue temperature [24]. The paper quantifies the expected yield reductions for each degree Celsius increase in global mean temperature; for instance, wheat, rice, maize, and soybean yields are reduced by 6.0%, 3.2%, 7.4%, and 3.1%, respectively. These estimates underscore the varying sensitivity of different crops to temperature changes [19]. The study shows that rice yields decrease with higher night temperatures linked to global warming, with a 10% decline in yield for every 1°C rise in growing-season minimum temperature. This highlights the need to consider the differential effects of day and night temperatures, as they have been shown to negatively impact rice yields [25]. The study found that droughts reduced harvested area and yields, while extreme heat primarily affected yields. Maize was more susceptible to extreme heat, causing an 11.7% yield deficit. Recent droughts had a 7% increase in damage compared to earlier ones, suggesting that the effects of extreme weather may be worsening over time [26].

Increased soil temperature also influences soil microbial activity, which can have variable effects on nutrient cycling [27, 28]. Soil organic carbon, enzyme activity, and microbial community responses to short-term drought and N application vary, indicating they impact soil health and microbial dynamics. Drought stimulates microbial activity, crucial for soil nutrient cycling [29, 30]. Studies focusing on different agroecological zones have demonstrated the differential responses of soil N mineralization rates to elevated temperatures [27, 31]. Moreover, higher temperatures directly impact crop physiology, such as thermal stress, altered enzyme activity, and protein denaturation, leading to faster growth cycles, which can reduce overall biomass accumulation and yields if water or nutrient availability is limited [25, 32]. The study highlights the importance of temperature during vernalization and ripening for optimal wheat development, impacting yield and quality. While precipitation is crucial for wheat yield, temperature also plays a significant role, with the interaction between temperature and water availability being crucial [33]. Rising temperatures can directly affect root development by increasing soil temperatures. This can lead to changes in root architecture, such as increased root elongation and altered branching patterns. For instance, elevated temperatures can enhance root auxin content, promoting root elongation and reorientation [34].

#### 3.1.2. Impact of Alterations on Precipitation Patterns

Changes in rainfall patterns, such as droughts and floods, disrupt nutrient cycling, reducing availability and affecting crop uptake efficiency. Drought conditions reduce water availability, while excessive rainfall leaches nutrients, further affecting crop production. These patterns also make it harder for crops to access necessary nutrients due to limited water availability and flooding conditions [26, 35]. Consequently, changes in nutrient availability combined with altered temperature and water availability often result in reduced yields and overall crop quality [19]. The study found that precipitation patterns during critical growth phases, especially during flowering and grain filling, significantly impact grain yield and protein formation in winter wheat. It concluded that an adequate water supply is crucial for maximizing wheat crop yield and quality [33].

Drought causes soil microorganisms to decrease growth and respiration rates due to limited water availability and increased osmotic stress, resulting in decreased carbon release into the atmosphere. Rewet soils experience the “birch effect,” where CO_2_ emissions spike due to the sudden availability of moisture and nutrients, causing significant short-term carbon losses from the soil [36]. This is particularly relevant for less mobile nutrients like P and micronutrients. Excessive rainfall can lead to soil saturation and waterlogging, creating anaerobic conditions that inhibit microbial respiration, increase denitrification and the production of GHGs like N_2_O, and increase nutrient losses through leaching and runoff [37]. The study demonstrated that soils showed significant recovery in microbial processes after stress events ended, with most parameters returning to control levels. Drought conditions caused more severe changes in soil respiration rates and enzyme activities than flooding, suggesting drought may pose a greater threat to microbial activity in agricultural soils [28]. Prolonged droughts lead to the desiccation of the upper soil layers, reducing microbial activity and slowing nutrient transport toward plant roots [36]. The study demonstrated that drought affects soil bacterial composition depending on crop type, with corn plots experiencing decreased diversity and soybean plots experiencing reduced microbial biomass carbon. Microbial inoculation did not significantly improve plant growth or drought tolerance [38].

#### 3.1.3. Elevated CO_2_ Concentrations Impact on Crop Quality and Nutrient Availability

Plant species, particularly C3 plants, show a significant increase in photosynthesis rates and growth under elevated CO_2_ conditions, while C4 plants show little to no growth enhancement. C4 crops do not experience an increase in yield, unlike C3 crops like wheat, rice, and soybean, which show 12%–14% increases. C4 plants' less responsiveness to N and protein concentration changes may impact crop nutritional quality [39]. While increased atmospheric CO_2_ levels may enhance photosynthesis, they can also result in the “nutrient dilution effect,” where plant tissue concentrations of essential nutrients decrease. Increased CO_2_ boosts grain yield and biomass in many crop species, but it often lowers grain nutritional quality because of changed ion profiles, particularly lower iron and zinc concentrations, and also N and protein content of cereal crop seeds [34, 40]. The concentration of CO_2_ and N_2_O in the atmosphere interacts with various soil properties through complex biological and chemical processes, influencing nutrient dynamics, microbial communities, and overall soil health [37]. Climate change will cause microbial community changes, impacting interkingdom interactions, biogeochemical cycling, and carbon flow. Studying soil microbiomes is challenging due to direct and indirect effects, such as elevated CO_2_ influencing plant rhizodeposition and soil moisture content [41]. Elevated CO_2_ negatively affects plant mineral status by reducing nutrient acquisition and assimilation efficiency, not due to impaired root architecture but the downregulation of membrane transporters involved in nutrient uptake, posing threats to crop quality, nutrient cycles, and terrestrial agroecosystems [42]. The study suggests that increased CO_2_ can improve water use efficiency (WUE) by reducing plant transpiration rates, but it could also negatively impact agricultural productivity due to higher temperatures and changed precipitation patterns, and could affect the availability of essential nutrients like potassium (K), P, and magnesium [43].

### 3.2. Limitations of Conventional Nutrient Management Under Climate Change

**Figure 3:**
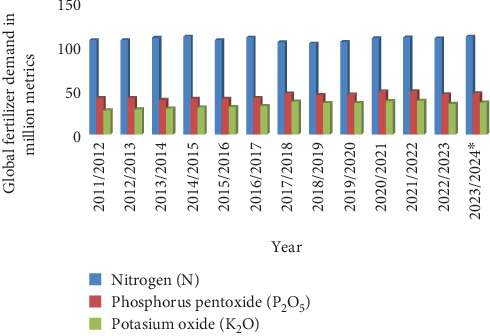
Global demand for agricultural fertilizer by nutrient from 2011/2012 to 2023/2024 (in million metric tons). *Source:* IFA [44].

Conventional nutrient management practices largely rely on the application of inorganic fertilizers, particularly NPK, to meet crop nutrient needs (Figure 3). While these fertilizers have significantly contributed to increased crop yields in the past, their indiscriminate and excessive application has resulted in substantial environmental consequences [45]. Jayaraman et al. [46] highlight that conventional practices often lead to nutrient losses, which can be exacerbated by extreme weather events. The use of synthetic fertilizers has led to soil degradation, resulting in the depletion of organic matter and disruption of soil microbial communities [47]. High applications of N fertilizers contribute significantly to GHG emissions, exacerbating climate change [48]. Synthetic N fertilizers, particularly nitrate, significantly contribute to N pollution, affecting air and water quality, human health, and aquatic ecosystems. Moreover, the effectiveness of chemical fertilizers can be compromised by extreme weather events. Heavy rainfall can lead to fertilizer runoff, reducing NUE and causing environmental damage [49]; on the other hand, drought conditions can limit the solubility and mobility of fertilizer nutrients, hindering plant uptake [35]. These limitations highlight the need for more sustainable and resilient approaches to nutrient management. As Roy et al. [50] indicated, when P is applied to crops, the response can be divided into direct and residual effects. For instance, in a single-year rotation, 63% of the response was attributed to direct effects, while 37% was due to residual effects. In another scenario, where P was added to a rainy season cereal, the total response was 57% direct and 43% residual. This highlights the importance of considering both effects in nutrient management strategies. Proper nutrient management strategies are crucial for improving crop productivity and WUE, especially in water-scarce areas [51].

The critical role of balanced nutrient application in mitigating the negative impacts of excessive fertilizer use has been linked to environmental degradation and reduced nutrient-use efficiency. It advocates for a shift from overreliance on chemical fertilizers to a more integrated approach that includes organic sources (Paramesh, Mohan Kumar, et al., 2023b). The study reveals that N fertilizer application, atmospheric N deposition, and climate change significantly influence soil nitrous oxide emissions. The modeling approach provides a comprehensive understanding of these factors' interactions. However, uncertainties in simulated emissions arise from model parameterization and N-related processes, highlighting the need for improved data and modeling techniques [52].

### 3.3. Emerging Nutrient Management Strategies for Sustainable Crop Production

#### 3.3.1. Minimizing Nutrient Losses by Enhancing Fertilizer Efficiency

The inefficiency of traditional fertilizers, particularly in terms of N losses through volatilization, leaching, and denitrification, has been a major driver for the development of enhanced fertilizer efficiency. These fertilizers aim to synchronize nutrient release with plant uptake, thereby reducing losses and improving NUE. The findings of Tedone et al. [31] showed that optimizing the timing and splitting of N applications led to better nitrogen agronomic efficiency (NAE), which is crucial for sustainable wheat production.

*Slow-release fertilizers* (SRFs) are designed to release nutrients gradually over time. They are often coated with polymers or other materials that control the rate of nutrient dissolution. The benefits are reducing N losses and increasing crop yields, particularly in areas with high rainfall or sandy soils [53]. According to Noulas et al.'s [54] report, polymer-coated fertilizers can raise phosphate fertilization efficiencies and crop yields. This is particularly important in the context of increasing global food demand and the need for sustainable agricultural practices. Y. Wang et al. [30] suggested using coated N fertilizers and multiple N applications to boost yield, grain protein content, and water productivity. It also emphasizes timing, recommending preanthesis N addition in medium soil textures and humid climates to optimize farmers' N management practices. The application of organic fertilizers in combination with inorganic fertilizers significantly enhanced fertilizer use efficiency. Specifically, the combination of recommended doses of fertilizers (RDFs)+vermicompost (VC) at 2.5 t/ha + FYM at 5 t/ha + *Azotobacter* resulted in maximum values for various efficiency metrics, including partial factor productivity (PFP), agronomic efficiency (AE), recovery efficiency (RE), and physiological efficiency (PE) [13].

Panhwar et al. [11] stated that P plays a crucial role in wheat nutrition, highlighting that imbalanced fertilization is a major factor leading to low fertilizer use efficiency. The proper timing and application methods, such as banding P with top dressing and sulfuric acid, can significantly enhance NUE in wheat crops. Goulding et al. [55] emphasize the significance of enhancing NUE in various farming systems, highlighting the potential benefits of cover cropping while also addressing potential trade-offs like increased nitrate leaching for sustainable practices. Strategies to improve NUE include precise fertilizer application techniques and the use of SRFs to minimize leaching and runoff.

*Controlled-release fertilizers* (CRFs) are more advanced than SRFs and provide more precise control over nutrient release. CRFs are often based on more advanced polymeric or inorganic coatings and are designed for specific crop needs based on growth stages, temperature, soil moisture, and pH. CRFs and coating technologies like sulfur and polymer are enhancing efficiency and environmental compatibility, while nanofertilizers coated with biodegradable materials improve crop resilience [56]. The adoption of CRFs is mentioned as a strategy to optimize nutrient availability and uptake efficiency. This method helps in reducing nutrient losses and improving the sustainability of nutrient management practices [57]. The highest rates of N fertilizer led to increased GHG emissions and energy consumption, with little to no significant increase in grain yield. Conversely, the optimized split application strategy not only improved yield and quality but also reduced GHG emissions and energy use [31]. The findings of Zhang et al. [7] suggested that INM can lead to improved NUE by optimizing nutrient inputs and matching nutrient supply with crop requirements. For instance, the implementation of split fertilizer applications and better management practices has been shown to reduce N fertilizer inputs by 24% while increasing yields by 12% across various sites. The application of two-thirds of the N fertilizer at later growth stages (tillering and stem elongation) was particularly beneficial. This approach maximized yield and quality while minimizing environmental impacts, suggesting a promising strategy for sustainable wheat production [31]. The implementation of SSNM significantly improved rice and maize yields by 24% and 69%, respectively, compared to traditional farmer practices, and when compared to local blanket fertilizer recommendations, yield improvements were 11% for rice and 4% for maize [58]. Innovations in fertilizers, such as SRF and CRF, significantly contribute to nutrient management; this helps in reducing nutrient losses and improving the efficiency of nutrient uptake by plants, which is essential for sustainable agriculture [59]. The study highlights that in-season root-zone N management strategy enhances N use efficiency, aligns N application with crop needs, and reduces N losses, contributing to sustainable maize production practices and high yields [60].

*Stabilized fertilizers* are products that contain additives, such as urease inhibitors or nitrification inhibitors, that slow down the process of N conversion in the soil, thus minimizing gaseous losses of ammonia or nitrous oxide. The effectiveness of stabilized N fertilizers in reducing GHG emissions and improving N availability for crops was demonstrated by Suter et al. [61].

#### 3.3.2. Tailoring Nutrient Application in Precision Agriculture

Precision nutrient management (PNM) practices are essential for optimizing crop yields while minimizing environmental impacts. This approach tailors nutrient applications to the specific needs of crops based on various factors, including soil characteristics, crop requirements, and environmental conditions, particularly through the lens of SSNM and the use of decision support tools like Nutrient Expert (NE) [62]. PNM tools like optical sensors, chlorophyll meters, leaf color charts (LCCs), and crop models improve NUE, leading to higher crop yields. The SPAD meter, LCC, and Green Seeker optical sensor are effective for N management [63]. The study emphasizes the importance of effective nutrient management in climate-smart agriculture (CSA), particularly SSNM, which optimizes crop yields, reduces GHG emissions, and enhances soil health, integrating precision farming technologies like remote sensing and GPS [64]. As Erickson and Fausti [65] reported, precision agriculture can significantly enhance the sustainability of production agriculture. By applying fertilizers and pesticides only where and when they are needed, PA can reduce environmental loading, thereby contributing to long-term sustainability in farming practices.

Advanced technologies like PNM and unmanned aerial vehicle–based spraying can minimize the negative effects of nutrient application, optimizing durum wheat productivity by considering timing, rate, and placement, enhancing grain yield and protein content while minimizing environmental impacts [57]. There is a need for tailored fertilizer management strategies in different regions, particularly in developing countries, emphasizing the importance of optimizing nutrient use for cereal crops that dominate fertilizer application and addressing micronutrient deficiencies [66]. The implementation of PNM strategies positively influenced soil enzymatic activity, which is crucial for nutrient cycling and soil health. The study highlighted that these strategies helped in modulating enzymatic activity, thereby improving soil biological and chemical parameters [67].

Nayak et al. [2] suggest that incorporating innovative technologies like soil testing and real-time nutrient management tools can significantly improve nutrient application practices, align with the “4R” principles, and enhance crop yields. PNM practices can enhance wheat cultivation NUE, optimize input costs, and reduce environmental impacts due to excessive fertilizer use [67]. The PNM techniques were found to enhance root morphological parameters, including root length density and surface area density, and robust vegetative growth, leading to an increased leaf area index (LAI) and improved solar radiation capture. This improvement in root structure facilitates better nutrient uptake, provides a balanced nutrient supply to crops, and contributes to overall plant health and productivity [67].

*Variable-rate application* (VRA) is a system that uses sensors, GPS, and data analysis tools to map spatial variations in soil nutrient levels and crop health. Based on these maps, fertilizer application rates are adjusted across the field to match site-specific nutrient requirements. Research has shown that VRA can significantly reduce fertilizer inputs while maintaining or even increasing crop yields [68]. SSNM is a strategic approach that aims to provide nutrients to crops based on their specific needs at different growth stages. SSNM can significantly improve crop yields and WUE by ensuring that nutrients are applied in the right amounts and at the right times, tailored to the specific conditions of each field. Jat et al.'s [62] study showed that SSNM practices led to a mean increase in system productivity, WUE, and net returns by 13.4%, 13.3%, and 15.3%, respectively, compared to the farmer's fertilizer practice (FFP). Additionally, the mean grain yield was 5.3% and 10.5% higher with RDFs and SSNM compared to FFP, respectively. This indicates that both improved tillage and nutrient management practices can lead to better crop performance and yield. Studies have shown that following SSNM recommendations can lead to significant improvements in grain yield and N use efficiency. For instance, N use efficiency was found to be 28% higher with SSNM compared to farmer practices, indicating that SSNM not only improves nutrient efficiency but also contributes to greater profitability for farmers [58].

*Remote sensing and proximal sensing*: Remote sensing technologies, including satellite and aerial imagery, are increasingly used to monitor crop health and nutrient status, providing valuable information for PNM. Proximal sensing, using ground-based sensors, offers high-resolution data on soil properties and plant traits. These tools enable real-time decision-making for fertilizer application. Studies have highlighted the effectiveness of these technologies in optimizing nutrient application and maximizing NUE [69]. The study of Dobermann [66] suggests that implementing tailored field and farm-scale best management practices (FBMPs) can enhance NUE by optimizing nutrient application based on local biophysical and socioeconomic conditions. The study emphasizes the potential of digital tools like RiceAdvice and NE in disseminating SSNM recommendations, thereby enhancing the scalability and impact of these practices among a larger number of farmers [58]. Advanced technologies like optical sensors and NE software are used in SSNM practices to improve NUE, thereby enhancing crop yields [3].

*Decision support systems* (DSSs) are tools that use data from various sources, like soil analyses, weather forecasts, and crop growth models, to provide nutrient management recommendations. They help farmers optimize fertilizer application rates and timing, leading to better nutrient utilization and reduced environmental impact. The DSS for nutrient management in crops is a versatile tool that can be applied to various crops like rice, rapeseed, wheat, groundnut, maize, and potato. It offers personalized recommendations for fertilizer application based on specific parameters like soil test values, crop type, variety, sowing season, soil type, and targeted yield, ensuring accurate nutrient needs [70]. The use of sensors for real-time nutrient measurement can significantly improve NUE in conventional farming. This technology can drive DSSs that optimize nutrient applications, thereby enhancing nutrient uptake and overall farm productivity [55]. The study highlights that a real-time N management method is particularly effective for crops like rice and maize. It emphasizes the importance of a combined approach that includes preventive measures against N deficiency, utilizing tools such as the LCC, SPAD, or optical sensors to control N fixation effectively [3, 71].

**Figure 4:**
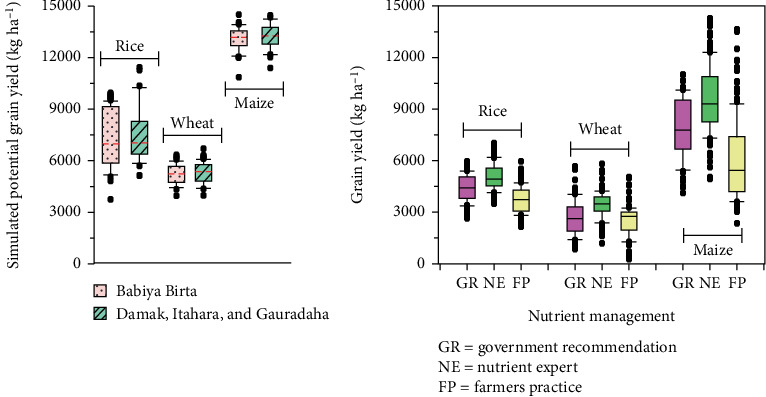
Climatic potential yield and yield under three nutrient management practices. *Source:* Timsina et al. [5].

The use of random forest (RF) models revealed that grain N uptake was the most critical factor for rice yields, while K uptake was most important for wheat and maize (Figure 4). This suggests that tailored nutrient management strategies can address specific crop needs effectively [5]. The NE tool for fertilizer recommendations significantly increased yields compared to both FFP and government recommendations (GRs) (Figure 4). Specifically, NE-based recommendations led to yield increases of approximately 3.5 t ha^−1^ for maize, 1.4 t ha^−1^ for wheat, and 1.3 t ha^−1^ for rice [5].

#### 3.3.3. Harnessing Natural Processes for Soil Health

Conservation agriculture (CA) practices, which include improved tillage methods, crop establishment techniques, and residue management, are critical for farmers to achieve sustainable crop production, better resource use efficiency, and a safe environment [62]. Parihar et al.'s [72] reports establish a comprehensive framework for nutrient management in CA systems, emphasizing the need to transition from conventional tillage practices and integrate nutrient management with CA's core principles of minimum tillage, residue mulching, and crop rotation for improved soil health and crop productivity. CA practices contribute to significant environmental benefits, including carbon sequestration, improved water quality, reduced soil erosion, and enhanced biodiversity, making it a climate-smart and sustainable agricultural approach [73]. The reviews highlight that CA is a global trend promoting sustainable alternatives to conventional farming, driven by farmer-led initiatives and increased stakeholder awareness. Jat et al. [62] suggest that adopting CA practices and improved nutrient management can lead to long-term sustainability in agricultural systems, especially in regions facing water scarcity and declining groundwater levels. CA's principles of minimal soil disturbance, permanent soil cover, and crop diversification promote healthy ecosystems, addressing issues like soil degradation and biodiversity loss [74].

Cover crops like legumes fix atmospheric N, enriching soil with essential nutrients. Crop rotation breaks pest and disease cycles, improving soil health and productivity. These practices reduce the need for chemical fertilizers. Integrating short-duration pulse crops like mungbean into the maize-wheat system improves soil health, nutrient availability, and overall system productivity [62]. Nakachew et al. [9] advocate for long-term sustainable practices like crop rotation and conservation tillage to improve soil health and reduce reliance on industrial fertilizers, contributing to the broader discourse on sustainable agriculture for food security and environmental health. Low yields in CA systems are often due to inefficient nutrient management, especially in degraded soils. To improve the initial success of CA practices, it may be necessary to grow green manure or legume crops for 1–2 seasons to build soil fertility before regular cropping begins [72]. The profitability of various nutrient management strategies is not solely determined by yield increase. N and P additions often result in low profit due to high fertilizer costs. Incorporating legumes in rotation leads to a 43% yield improvement and a net profit of $146–$263 per hectare [75]. The study highlights the importance of rye cover crops in enhancing soil carbon and N levels, demonstrating that incorporating cover crops into cropping systems can significantly increase crop residue, thereby improving soil quality [76]. Studies have consistently shown the benefits of cover cropping in increasing soil organic matter, improving nutrient availability, and reducing the need for synthetic fertilizers [2].

No-till farming, or zero tillage, promotes soil structure, water infiltration, and nutrient cycling, improving soil health, increasing carbon sequestration, and reducing nutrient losses, particularly in conservation agricultural systems [74]. The study reveals that using poultry litter in no-till systems not only improves soil carbon and N sequestration but also reduces environmental contamination from N leaching and P runoff, thus enhancing soil health [76].

The use of compost, manure, and other organic materials enhances crop growth, soil structure, water retention, and microbial activity, contributing to long-term soil health and improving nutrient cycling, especially in degraded soils, through nutrient amendments. The review emphasizes the importance of organic farming, which avoids synthetic chemicals and instead employs manual, mechanical, cultural, and biological strategies that are crucial in addressing the rising biotic stresses caused by climate change [77]. Organic farming integrates cultural, biological, and mechanical practices to preserve natural resources, biodiversity, animal welfare, and human health, enhancing food system resilience and promoting health, ecology, fairness, and care [78].

#### 3.3.4. Harnessing Microbes for Biofertilizers

Biofertilizers are natural products that use beneficial microorganisms to improve nutrient availability and promote plant growth, providing a sustainable alternative to synthetic fertilizers. According to Panhwar et al. [11], the application of biofertilizers can lead to a significant increase in grain yield while reducing the need for N fertilizers. This not only benefits growers by lowering input costs but also contributes to environmental sustainability by minimizing N oxide emissions associated with chemical fertilizers. Bargaz et al. [79] conclude that continuous research and development of microbial-based biofertilizers and biostimulants are necessary to enhance crop yield and resilience to environmental changes. This will contribute to sustainable agricultural systems capable of meeting future food production needs. N-fixing bacteria like *Rhizobium* and *Azotobacter* convert atmospheric N into plant-available forms, reducing the need for N fertilizers, particularly in legume crops. Research on these bacteria has expanded from pure research to wide-scale industrial applications, with the potential to reduce the need for N fertilizers. Kantwa et al. [80] reported the importance of integrating biological inoculants like *Azotobacter* and phosphate solubilizing bacteria (PSBs) with chemical fertilizers for improved nutrient management and crop yields, particularly in regions facing soil health issues.

Roy et al. [50] underscore the need for balanced nutrient management to maintain soil fertility and prevent environmental hazards, particularly the contribution of N from various sources, including biological N fixation and recycling from crop residues. This information is crucial for understanding the sustainability of nutrient use in agriculture. Gojon et al. [42] suggested that increasing N supply to plants can alleviate the negative effects of elevated CO_2_, helping to recover the potential increases in photosynthesis and biomass production that the “CO_2_ fertilization” effect could provide. Mycorrhizal fungi form symbiotic associations with plant roots, enhancing nutrient uptake, particularly P and micronutrients. Studies have demonstrated that mycorrhizal biofertilizers can improve plant growth, especially in P-deficient soils [81, 82].

Phosphate-solubilizing microbes can solubilize inorganic P in the soil, making it more available to plants and reducing reliance on phosphate fertilizers. It can improve soil carbon reserves and enhance the availability of fixed soil P through beneficial microbial activity [11]. The study suggests that bacteria like *Acinetobacter* and *Pseudomonas* can produce gluconic acid, boosting plant growth and soil P availability, thereby improving crop yield and reducing chemical fertilizer use, thus promoting sustainable agriculture [83]. Phosphate-solubilizing microorganisms can be used as biofertilizers, crucial in sustainable agriculture, enhancing P availability, promoting long-term soil health and productivity, and reducing reliance on chemical fertilizers [83, 84].

#### 3.3.5. Adaptation Strategies in Nutrient Management Under Climate Change

CSA practices, including improved crop varieties, efficient water management, and integrated soil fertility management, have been proven to increase crop yields, especially in grain crops like rice, maize, and wheat, according to several studies, providing practical insights for farmers and policymakers [85]. The application of organic amendments significantly boosts the wheat photosynthetic rate, leading to increased biomass and grain yield, emphasizing the importance of nutrient management in crop performance optimization [86]. The study found that combining organic amendments with the recommended inorganic fertilizer dose significantly increased wheat grain yield by 27.01% compared to the control (NPK) treatment. The highest yield was achieved with NPK+*Azolla* compost, followed by cow dung, rice husk dust, and green manure, indicating the effectiveness of organic amendments [86]. The study by Garnaik et al. [87] suggests that the continuous use of high doses of inorganic fertilizers without organic amendments may not support long-term productivity, recommending the adoption of balanced doses of NPK with FYM for high productivity and sustainability in rice–rice systems. The proposed nutrient management strategy includes a balanced blend of synthetic, organic, biochar, and bioorganic fertilizers, aiming to enhance nutrient cycling, water conservation, and biodiversity preservation, crucial for sustainable agricultural practices [88]. Soil health is crucial for sustainable agriculture, and practices like biochar, VC, and bio-enriched rock phosphate enhance organic carbon levels, soil structure, and microbial activity, thereby enhancing resilience against climate-induced stresses [17]. Agroecological nutrient management emphasizes the need for a balanced approach that integrates organic and biofertilizers with traditional farming practices to optimize yields while maintaining environmental health [88]. The advantages of combining legumes and grains in intercropping systems open the door for creative farming methods that can address the twin issues of environmental sustainability and food security. This study has broad ramifications and offers a solid foundation for future farming practices in areas with comparable soil and climate circumstances [89]. The study demonstrates that adopting CSA, specifically the combination of moderate deficit irrigation and adequate nutrients, resulted in improved energy use efficiency (6.24), carbon efficiency (16.02), and overall biomass yield (13.29 t ha^−1^), indicating the potential for reducing energy consumption and carbon footprints in agriculture [4].

Organic farming enhances soil health and reduces chemical runoff, while INM promotes resource efficiency and minimizes the carbon footprint associated with synthetic fertilizer production, and both contribute positively to environmental sustainability [90]. Biochar is a valuable soil amendment that improves soil quality, enhances nutrient availability, and boosts crop yields. It can mitigate climate change and promote food security, although its effectiveness depends on the varying nutrient compositions of different types of biochar [91]. The study underscores that agriculture has significant potential to mitigate GHG emissions through practices such as carbon sequestration in soil and reducing methane and nitrous oxide emissions. However, the adoption of these practices is currently limited due to various challenges, including a lack of awareness and confidence in monitoring [22]. The management of crop residues is highlighted as a vital component of nutrient management strategies. Retaining crop residues on the soil surface can enhance soil organic matter, improve moisture retention, and provide a habitat for beneficial soil organisms. However, the burning of residues, while sometimes economically beneficial in the short term, can lead to long-term soil degradation and nutrient loss, highlighting the need for sustainable residue management practices [46]. Microorganisms manage drought by utilizing osmoregulation, which involves synthesizing compatible solutes like trehalose and proline to maintain cellular integrity and hydration. Fungal communities are more resistant and resilient than bacterial ones, suggesting they may have intrinsic adaptations that enable them to grow more effectively at lower moisture levels [36]. The study reveals that microbial community resistance and resilience are influenced by specific functional traits, with the abundance of r-strategists and K-strategists playing a crucial role in determining responses to climate change-related disturbances [92].

The review of Zheng et al. [85] indicated that CSA adoption contributes to lowering GHG emissions, including CO_2_, N_2_O, and CH_4_. This environmental benefit is crucial for addressing climate change and promoting sustainable agricultural practices. The development and dissemination of safe and effective climate-smart fertilizers are crucial for achieving higher crop yields and enhancing resilience against climate change. This indicates that innovative fertilizers can play a pivotal role in addressing food security challenges [59].

The challenges faced in implementing CSA practices at scale include the lack of proper knowledge and streamlined policy frameworks. It calls for collaboration among policymakers, researchers, and industry stakeholders to overcome these barriers and promote the adoption of climate-smart fertilizers [59]. The study reveals that temperature impacts crop and maize revenues negatively but positively affects tea revenues. It suggests farmers should adopt integrated approaches, including drought-tolerant crop varieties and sustainable farm management, to enhance resilience against climate variability [93]. CSA practices mitigate production risks and reduce vulnerability to climate-related shocks that enhance food consumption, dietary diversity, and food security, which are crucial for adapting to climate change. It helps farmers better cope with adverse climate events, thereby reinforcing the resilience of agrifood systems [85]. Linking farmer research with academic insights enhances CSA effectiveness, promotes policy promotion, and empowers farmers. Engaging farmers as active researchers fosters adaptation strategies and collective knowledge [94].

#### 3.3.6. A Holistic Approach to INM

**Figure 5:**
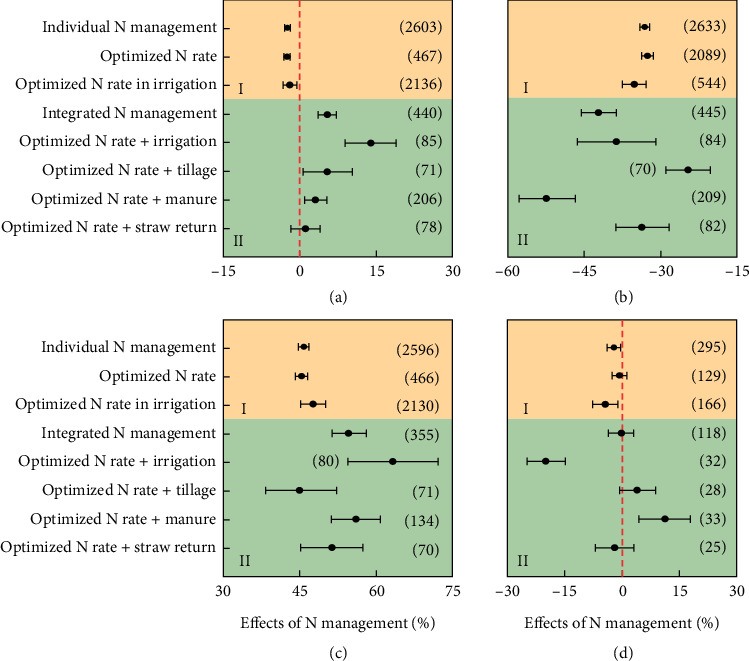
Effects of N management practices on (a) grain yield, (b) N application rate, (c) NUE, and (d) WUE. The number of observations included in each category is shown in parentheses. I (at the top) represents individual N management practice, and II (at the bottom) represents integrated N management practice, with error bars representing 95% confidence intervals. *Source:* Liu et al. [96].

INM is a comprehensive approach that integrates organic and inorganic nutrient sources, biofertilizers, and soil health practices to optimize nutrient availability, enhance crop yields, reduce chemical fertilizer dependency, minimize environmental impact, and ensure food security [95]. INM systems, designed for specific site conditions, crop requirements, and resources, integrate biofertilizers with organic and inorganic amendments, reducing chemical fertilizer use while maintaining or increasing wheat yields and promoting sustainable agricultural practices. Jat et al. [12] reported the importance of combining organic and inorganic nutrient sources to optimize soil health and crop productivity, but they argue that neither chemical fertilizers nor organic manures alone can sustain productivity, advocating for a balanced approach (Figure 5). INM practices can minimize nutrient losses, particularly in intensive agricultural systems, thereby ensuring that crops receive the necessary nutrients for optimal growth and maintaining high nutrient-use efficiency [97]. Liu et al.'s [96] study reveals that integrated N management practices, which combine optimized application rates with other agronomic practices, improve P and WUE, leading to improved wheat yields and N use efficiency. These practices not only increase yield and NUE but also demonstrate better stress tolerance across soil-climatic conditions (Figure 5). As reported by Parewa et al. [14], the economic evaluation revealed that the highest net returns were achieved with the application of 75% NPK combined with 10 t ha^−1^ FYM and PGPR (plant growth–promoting rhizobacteria) combined with VAM (vesicular arbuscular mycorrhiza), yielding a B:C ratio of 2.37. In contrast, 100% NPK alone resulted in a lower B:C ratio of 2.31. This indicates that integrating organic and inorganic inputs can lead to better economic outcomes for farmers. The combination of organic and inorganic nutrient sources, such as FYM and biostimulants, leads to improved soil health and higher grain yields. The findings indicate that wheat crops treated with INM practices achieved significantly better growth and dry matter accumulation compared to control groups [15]. The application of a 100% RDF combined with *Azotobacter* and PSB led to significantly higher nutrient content in both grain and straw, improved nutrient uptake by the plants, and the soil's available NPK levels [80].

Zhang et al. [7] reported that due to the introduction of INM as a primary nutrient management strategy, there has been a notable decrease in fertilizer application rates. For instance, in areas where INM was practiced, fertilizer consumption has steadily declined since 2004, while the PFP for chemical fertilizers has increased from 17 kg/kg in 2004 to about 21 kg/kg in 2008. Integrated N management practices led to an average increase in wheat yield by 5.4% and improved NUE by 55%, whereas solo application of N decreased wheat yield by 2.4% and improved NUE by 45%. This indicates that the crops were able to utilize N more effectively compared to individual practices and increased yield [96]. The combination of NPK with FYM significantly enhanced rice yield by 34%–38% compared to chemical fertilizer alone in both wet and dry seasons and also improved energy use efficiency by 13.6% and 20%, respectively [87, 98]. The findings by Zhang et al. [7] demonstrated that INM is crucial to the optimized N rates using in-season root-zone management, not only reducing N fertilizer use by 60% for wheat and 40% for maize but also increasing grain yields by 4% and 5%, respectively. This indicates that INM can be both economically and agronomically beneficial. The combined application of the RDF, VC, FYM, and *Azotobacter* produced the highest grain yield compared to other treatments. This treatment resulted in a notable increase in yield, leading to higher NPK uptake by the wheat crop [13]. The study by Ejigu et al. [99] found that the combination of compost and mineral fertilizers significantly enhanced soil pH, organic carbon, total N, P, sulfur, and cation exchange capacity, demonstrating the effectiveness of INM.

INM is a crucial strategy for enhancing sustainability in the Indian rice–wheat cropping system, emphasizing the need for a balanced approach to managing nutrient levels [100]. The study reveals that nutrient application rates alone do not fully explain yield improvements in sorghum. High relative yield improvements were achieved under integrated nutrient application rates, suggesting an optimal range for maximizing yield without excessive costs [75]. The research also shows that integrated fertilizer application leads to higher maize yields than mineral fertilizers alone, with the highest maize dry biomass and grain yield recorded [99]. Organic farming uses natural fertilizers, like compost and manure, which can cause nutrient imbalances, impacting crop growth and yield. INM addresses these demands, enhancing crop yields [90] while minimizing environmental impacts [54]. The findings of Goulding et al. [55] suggest that integrated farming systems are more adaptable to changing agricultural needs, requiring expertise from land managers for effective NUE and sustainable farming practices. Developing innovative integrated plant nutrient management systems (IPNMS), combining microbial and mineral resources, promotes sustainable agricultural practices, increasing ecoefficiency in fertilizer use and ensuring long-term productivity [79]. INM, a strategy that combines organic and inorganic fertilizers, is crucial for improving soil fertility and crop productivity, especially beneficial for resource-poor farmers with limited access to chemical fertilizers [46].

Effective nutrient management is crucial for pasture-based dairy systems' sustainability, as well-managed pastures can recycle nutrients, enhance soil organic carbon buildup, and aid carbon sequestration, while poor management can lead to imbalances and reduced herbage production, reducing carbon storage potential [101]. The study reveals that combining organic and inorganic fertilizers enhances rice yield, nutrient efficiency, soil fertility, carbon sequestration, root growth, and water retention and contributes to climate change mitigation by improving soil properties and promoting root growth [102]. INM enhances soil organic matter, macro- and micronutrient levels, and health, with nitrate fertilizers being more effective than ammonium, and combining poultry manure with inorganic fertilizers yields better results [98]. INM not only increases nutrient uptake by plants but also enhances the physicochemical properties of the soil, such as soil organic carbon, microbial activity, and enzymatic activities, which are critical for upholding soil fertility and sustainability [15]. The study of Sharma et al. [100] suggests that INM is a viable option for the Indian rice–wheat cropping system, ensuring food security, soil health, and sustainability while meeting population growth needs.

Sharma et al. [100] highlight that INM practices contribute positively to soil health and environmental sustainability by reducing the reliance on synthetic fertilizers and improving nutrient recycling within the soil–plant system. Sustainable nutrient management practices contribute to long-term soil health by replenishing nutrients and preventing soil degradation. INM, a sustainable agriculture model, integrates organic and inorganic nutrient sources, promoting high crop productivity while minimizing environmental impacts, making it a flexible approach [90]. The paper emphasizes the importance of INM strategies in addressing global food security challenges, particularly in meeting the 70% increase in food production by 2050 while preserving environmental sustainability [97].

### 3.4. Challenges and Future Prospects

Under climate change, achieving sustainable crop productivity is fraught with challenges that cut across the sociopolitical, economic, agronomic, and environmental spheres. Especially in vulnerable areas like South Asia and sub-Saharan Africa, these obstacles frequently compound one another, increasing their impact on food security. Climate-induced disruptions to soil nutrient cycles and health are a major concern. Increased temperatures accelerate soil organic matter decomposition, leading to faster nutrient mineralization but higher losses through volatilization and leaching. Droughts, floods, and soil erosion further deplete topsoil nutrients, affecting 33% of arable land. These disruptions challenge traditional fertilizer application models, leading to overfertilization and environmental harm. Nutrient mismanagement contributes to GHG emissions, with N fertilizers accounting for 5% of global anthropogenic nitrous oxide. P runoff from farms pollutes freshwater systems, and biodiversity loss reduces natural nutrient recycling. Balancing nutrient inputs while sustaining yields is difficult in intensive farming systems, and farmers lack access to real-time soil testing or climate forecasting tools. Precision agriculture technologies face adoption barriers due to high costs and technical complexity. Limited long-term data on climate-nutrient interactions in diverse agroecological zones hinders predictive modeling and leaves farmers without tailored advice.

Despite these challenges, innovative approaches offer promising pathways to enhance sustainability. Future prospects hinge on interdisciplinary integration, leveraging technology, biology, and policy to build resilient systems. Precision and digital agriculture, supported by artificial intelligence, IoT sensors, and remote sensing, are increasingly recognized for their capacity to enhance NUE by 10%–30%. Complementary tools such as drones, satellite imagery, and machine learning models allow real-time monitoring of soil health and predictive nutrient management, thereby reducing waste and supporting adaptive responses to climate variability. By 2030, such technologies could be particularly impactful in resource-limited regions where nutrient inefficiency remains a major barrier to productivity.

Beyond digital solutions, bio-based approaches, including biofertilizers and organic amendments, play a critical role in mitigating emissions while strengthening soil biodiversity. Circular economy strategies, such as recycling urban waste into fertilizers, provide sustainable alternatives to address P scarcity. Concurrently, advances in biotechnology, including genetic engineering for enhanced nutrient uptake and gene editing for stress tolerance, may mitigate the nutrient dilution effects associated with elevated CO_2_ and accelerate the development of resilient crop varieties.

**Figure 6:**
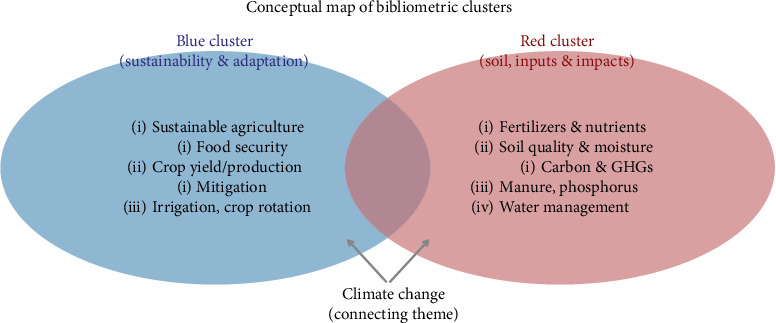
Conceptual map of bibliometric clusters.

Agroecological practices, such as crop rotation, intercropping, and agroforestry, further contribute by enhancing natural nutrient cycling and buffering systems against climate extremes (Figure 6). Integrating these practices within CSA frameworks, alongside early warning systems, strengthens resilience and adaptation capacity. Importantly, the success of these innovations depends on global cooperation through research investments, educational initiatives, farmer cooperatives, and equitable trade agreements, supported by AI-driven platforms that facilitate cross-border knowledge exchange and technology dissemination.

#### 3.4.1. Blue Cluster

The blue cluster highlights research on sustainable practices and productivity-oriented approaches to climate change and agriculture, focusing on crop yield, food security, soil fertility, irrigation, crop rotation, and smallholder mitigation and improving agricultural resilience through crop management, soil fertility, and precision agriculture. It reflects research on sustainable agriculture and adaptive strategies to climate change.

#### 3.4.2. Red Cluster

The red cluster explores the environmental impacts of agriculture, focusing on inputs, soil processes, and nutrient management. It highlights the impact of N, manure, carbon, P, soil quality, soil moisture, nutrients, carbon sequestration, and water management on soil and climate interactions. It also highlights the connection between fertilizer use, soil quality, and climate change, and the environmental consequences of agricultural inputs and soil nutrient cycles.

**Figure 7:**
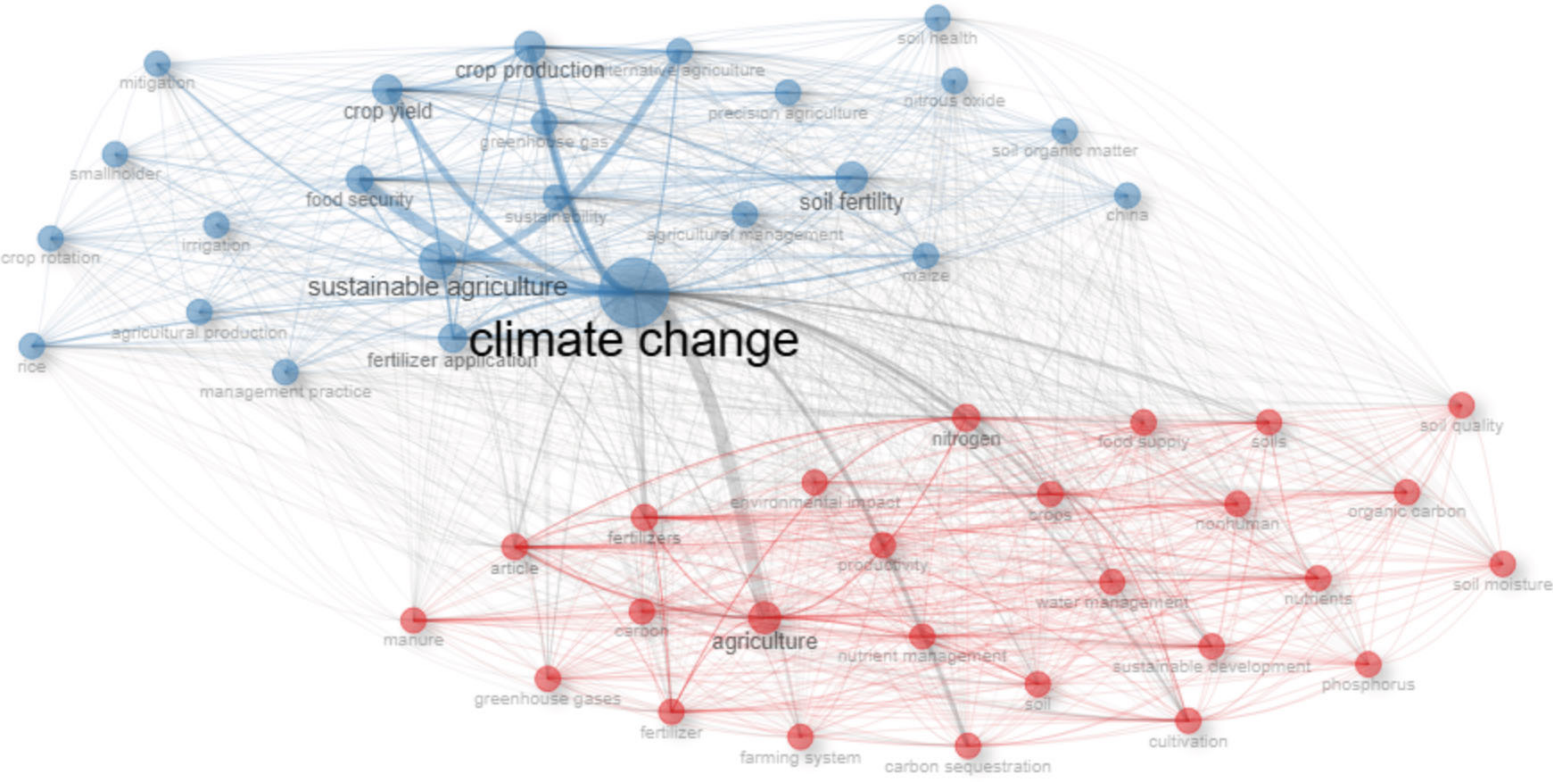
The analysis of the co-occurrence network of the Top 40 keywords using VOS clustering, with blue and red clustering results, connecting lines, and node sizes representing the frequency of keyword appearance in the article.

The center (climate change): the climate change research theme, which integrates both perspectives, serves as a strong bridging link (Figures 6 and 7).

## 4. Conclusion

Climate change presents profound challenges to global agriculture by disrupting nutrient dynamics, reducing crop yields, and degrading soil health through increased temperatures, altered precipitation patterns, and frequent extreme weather events. Traditional nutrient management relying heavily on chemical fertilizers is increasingly unsustainable, contributing to nutrient losses, environmental pollution, and GHG emissions. This highlights the urgent need for sustainable, efficient, and adaptive nutrient management practices.

This review emphasizes the need for sustainable, resilient nutrient management practices to mitigate environmental damage. The limitations of excessive inorganic fertilizer use, coupled with climate-induced disruptions in nutrient cycles and crop productivity, necessitate the adoption of strategies like enhanced fertilizer efficiency, precision agriculture, harnessing natural soil processes, and INM. It is increasingly evident that a combination of technologies, tailored to specific agroecological conditions, will be essential to effectively address the global challenge of feeding a growing population while mitigating the impacts of climate change. To tackle climate change, it is crucial to implement comprehensive mitigation and adaptation strategies, including sustainable agricultural practices, innovative technologies, and policy frameworks. These strategies should reduce GHG emissions and enhance resilience in agricultural systems. However, successful implementation requires overcoming knowledge gaps, policy frameworks, and farmer adoption, emphasizing the need for future research and practical implementation.

Ultimately, nutrient management must be viewed not as a standalone intervention but as a cornerstone of CSA. A holistic, systems-based approach, one that harmonizes AE, environmental stewardship, and socioeconomic viability, is essential for future-proofing global food systems. Future research should continue to focus on the development and evaluation of climate-smart nutrient management practices, understand the complex interactions of various climate stressors on nutrient cycling and crop productivity, and facilitate the adoption of effective and economically viable technologies by smallholder farmers. In addition, studies focused on social, economic, and policy considerations are critical to achieving a transition toward sustainable agriculture under rapidly changing climatic conditions.

## Figures and Tables

**Table 1:** Criteria for selection of articles in the review.

**Criteria**	**Included**	**Excluded**	**Reason**
Publication date	Since 1998	Before 1998	To gain insight into a wide range of thematic areas
Publication language	Articles in English	Articles in other languages	English language only
Publication theme	Articles on nutrient management strategy, sustainable agriculture, and climate change	Articles not relevant to the topic	To address the issue properly
Availability of articles	Open access	Non-open access	Requesting purchase
Types of articles	Peer-reviewed articles	Articles other than peer-reviewed	Availability and originality of the articles

## Data Availability

The data that support the findings of this study are available on request from the corresponding author. The data are not publicly available due to privacy or ethical restrictions.
